# The Management of Non-Muscle-Invasive Bladder Cancer in a Veteran Patient Population: Issues and Recommendations

**DOI:** 10.3390/curroncol31110493

**Published:** 2024-10-28

**Authors:** Jennifer Taylor, Sagar Patel, Krishnanath Gaitonde, Kirsten Greene, Joseph C. Liao, Glen McWilliams, Mark Sawyer, Florian Schroeck, Aly Alrabaa, Gal Saffati, Shane Kronstedt, Jeffrey Jones

**Affiliations:** 1 Operative Care Line, Urology Section, Michael E DeBakey Veteran Affairs Medical Center, Houston, TX 77030, USA; 2 Scott Department of Urology, Baylor College of Medicine, Houston, TX 77030, USA; 3 Department of Urology, Cincinnati VA Medical Center, University of Cincinnati Cancer Institute, Cincinnati, OH 45220, USA; 4 San Francisco VA Health Care System, San Francisco, CA 94121, USA; 5 Department of Urology, UCSF Medical Center, San Francisco, CA 94121, USA; 6 VA Palo Alto Health Care System, Palo Alto, CA 94304, USA; 7 Department of Urology, Stanford University School of Medicine, Palo Alto, CA 94304, USA; 8 James J. Peters Veterans Affairs Medical Center, Bronx, NY 10468, USA; 9 Department of Urology, Mount Sinai School of Medicine, Bronx, NY 10468, USA; 10 Rocky Mountain Regional VA Medical Center, Aurora, CO 80045, USA; 11 White River Junction VA Medical Center, White River Junction, VT 05009, USA; 12 Dartmouth Geisel School of Medicine, Hanover, NH 03755, USA; 13 College of Natural Sciences and Mathematics, University of Houston, Houston, TX 77004, USA

**Keywords:** bladder cancer, blue-light cystoscopy, guidelines, Veterans’ Health

## Abstract

The ability of the Veterans Health Administration System to care for veterans with bladder cancer is influenced by the increased complexity of both veterans and the system’s capacity to do so, which is determined by personnel and equipment allocation. Herein, we review the guidelines for bladder cancer management in the context of this population and highlight unique veteran characteristics that impact the delivery of bladder cancer care within the Veterans Health Administration System. There are opportunities for standardization and implementation, which can improve the quality of this care, and we summarize the questions for which coordinated research efforts may provide answers.

## 1. Introduction

Over 9 million veterans receive medical care within the Veterans Health Administration (VHA) system. Bladder cancer (BC) is the third most prevalent non-cutaneous cancer in veterans and thereby represents a significant healthcare burden to the VHA System (VHAS). Although clinical guidelines for the management of BC provide evidence-based consensus recommendations regarding many aspects of care, they still contain areas of ambiguity which can lead to individual interpretations and disparities in care. VHA medical centers face unique challenges due to the system’s capacity and patient characteristics, which can influence their adherence to the guidelines. This article explores these issues and outlines the roles that consistent equipment allocation and new technology can have in meeting the needs of this patient population, by providing better care, saving expenditures and time, and allowing more standardized care across the entire VHAS.

### 1.1. Patient Population in the VHAS

Sociodemographic and health status differences can pressure resources within the VHAS [1]. Veterans using the VHAS tend to be older and have lower median incomes than veterans who do not rely on the VHAS for medical care [2]. These differences are partly due to the design of the system, in which higher-income veterans are often not eligible for VA services. Compared to non-veterans, veterans are more likely to be male (92%, as estimated in 2014) and much older, with 75% being 55 or above compared to 34% in the general population [2]. Almost 20% of recent veterans are unemployed, and 25% of those employed are on a low income (<USD 21,000/year) [3]. Housing and employment insecurity are more prevalent because military-acquired work skills may not readily transfer to the civilian sector, and PTSD and substance dependence negatively impact job maintenance [3].

Using data from 27,008 VA patients from 2003 to 2013, a study examining the prevalence and outcomes of non-muscle-invasive bladder cancer (NMIBC) in the veteran population reported a 5-year overall survival rate of 68% and a 5-year bladder-cancer-specific survival of 98% [4]. The disparity between these survival rates may be attributed to the number of comorbidities associated with the veteran population. Veterans have a higher unadjusted prevalence rate of diagnosed chronic health conditions such as cancer, diabetes, and chronic obstructive pulmonary disease than non-veterans [2]. Adjusting for demographic characteristics (age and gender) reduces but does not eliminate the difference in disease rates [2]. Thus, multi-morbidity is more common among patients within the VHAS compared to the general population, especially with the added mental health conditions among younger patients [5]. About 25% of patients who access the VHAS have a mental health condition and 3.3% have PTSD [2]. Personnel deployed to Iraq or Afghanistan who reported combat exposure were more likely to initiate smoking or resume smoking than non-combatants [3]. From 2003 to 2007, the percentage of active smokers among male and female veterans was significantly higher than the general population for males (27% vs. 21%) and females (23% vs. 18%), respectively [6].

Among veterans, those in the VHAS face more challenges, as evidenced by data showing the unadjusted prevalence of cancer is 96% higher for veterans in the VHAS compared to non-VHA veterans [2]. Around 50,000 incident cancer cases are reported annually in the VA Central Cancer Registry (VACCR) [7]. In 2010, cancer of the urinary bladder (stages 1 to 4) was the sixth most common site (*n* = 1546, 3.4%) behind prostate (29.1%), lung/bronchus (17.8%), colon/rectum (8.2%), kidney/renal pelvis (3.8%), and skin melanoma (3.7%) [7]. These registry data excluded all (stage 0) non-invasive and in situ cases (1473); with the addition of these non-invasive cases, BC would comprise 6% of all reported cancer cases, behind only prostate and lung/bronchus in incidence [7].

Exposures during military service also present unique risks to veterans. The Veterans and Agent Orange committee of the National Academy of Sciences and Institute of Medicine concluded that there is “limited or suggestive evidence for an association of bladder cancer with exposure to chemicals of interest,” which include herbicides such as Agent Orange and Agent Blue. However, they have not received a service connection status for veterans with bladder cancer [8]. Agent Blue has not been widely studied, but its main component, arsenic, has been associated with lung and bladder cancers [9]. Similarly, exposure to contaminated water at Camp Lejeune was associated with elevated mortality and solid tumors, including kidney, esophageal, cervical, liver, and Hodgkin’s lymphoma tumors [10].

The higher comorbidity burden, creates additional risk for the use of general anesthesia and surgical procedures in this veteran population. The transurethral resection of bladder tumors (TURBT) often necessitates maintenance of anti-platelet or anti-thrombotic medications and may require general anesthesia with muscle paralysis, so the inherent anesthetic risks are still substantial. Many veterans have an ASA Physical Status Classification of 3 or 4, denoting severe systemic disease (class 3) which may be a threat to life (class 4). Table 1 compares the ASA scores for a cohort of the general population, the national population of genitourinary (GU) patients, and GU patients in one institution (Micheal E. DeBakey VAMC, Houston) [11].

### 1.2. Challenges in VHAS in the Management of Bladder Cancer

To date, the VHAS has 1243 healthcare facilities, including 1063 outpatient sites, serving 9 million veterans. In 2007, around 143 hospitals had cancer diagnostic and treatment capabilities and shared a national electronic medical record (EMR). Recently, the VHAS has been criticized for a substandard quality of care, with reported deficiencies in the access to care within the Phoenix VAMC. Yet, patients in the VHAS see more specialists and make more emergency room visits than other non-military-managed care patients [1]. Veterans waited longer for treatment, and their average length of hospital stay was longer; however, as veterans who use the VHAS have poorer health and a more significant number of co-morbidities, this difference leads to higher resource use [1].

Following a major restructuring program in the mid-1990s, care in VA facilities often outperforms non-VA institutions for many outcome measures. This has been attributed to centralized decision-making capabilities, a salaried physician workforce, educational programs, mature EMRs, and fixed budgets [12].

The centralized and relatively fixed resources of the VHAS do present some unique challenges. In 2020, the American Urological Association (AUA) guidelines for NMIBC provided recommendations for a greater standardization of care [13]. However, efforts to follow the guidelines can strain the available resources. For example, guidelines call for repeat resections to be carried out approximately six weeks after the first resection in high-risk cases.Still, in the VHAS, the urologic block time allocation for endoscopic procedures nationwide is insufficient at certain sites to schedule additional surgeries in the appropriate time frame. Despite access to community urologists through the Choice Act, the long-term financial burden and need to re-establish care for veterans in local clinics may delay diagnostic and therapeutic plans.

Access to specialized pathologists may be critical to depict and diagnose bladder pathology accurately [14]. However, since many VA institutions do not have access to an experienced genitourinary pathologist, there may be difficulties in appropriately diagnosing patients with variant histology. One study found that 21% (0–80%) of the pathology reports for VA institutions lacked at least one of the four pathology synoptic summary components explicitly recommended by the College of American Pathologists protocol: histology, grade, presence of muscularis propria, and microscopic extent [15]. Compared to other solid tumors diagnosed by non-VA institutions, their complete synoptic summaries for tumor specimens ranged from 85 to 96% [16].

Regarding resource allocation, flexible cystoscopy for bladder cancer surveillance is the most common urologic procedure performed in VA sites [17]. Their surveillance schedules are complex, and there is low adherence to those recommended in national guidelines [17]. No veteran-specific guidance or standard operating procedure (SOP) exists. Given the volume of bladder cancer managed in the VHAS, there is a great need and opportunity to examine the surveillance protocol and standardize it, using features in the EMR, to increase efficiency and cost savings and potentially improve patient care.

## 2. Screening and Evaluation of Hematuria

Before the 2020 AUA guidelines for hematuria workup, several areas of ambiguity lead to individual interpretations and inconsistencies in care. The integration of risk stratification for hematuria has since helped standardize clinical and diagnostic workups, reducing healthcare costs, enabling effective patient counseling, and ensuring the appropriate allocation of resources to populations at higher risk of malignancy. Within the AUA guidelines for the management of NMIBC, there remain areas of ambiguity which can lead to varied interpretations and inconsistencies in care [13].

### 2.1. Asymptomatic Microhematuria

Asymptomatic microhematuria (AMH) is the most common presenting symptom of bladder cancer and is a finding seen in the microscopic evaluation of urine sediment [18]. The presence of at least three red blood cells (RBCs) per high-powered field (HPF), in the absence of infection, should trigger a referral for urologic evaluation [18]. However, the prevalence of AMH is 9–18% in apparently normal individuals, and only 2.6% of cases of AMH lead to the diagnosis of a malignancy [13]. It has been debated whether the workup of every individual with a single urinalysis showing ≥3 RBCs/HPF is an inefficient use of resources, causing a significant burden on healthcare systems.

The updated 2020 AUA hematuria guidelines suggest that clinicians risk-stratify patients based on the quality of the RBCs in their urinalysis, age, smoking history, and genetic and co-morbidity risk factors. A repeat urinalysis within six months or cystoscopy with renal ultrasound is appropriate for low-risk patients. Low-risk patients with persistent positive microhematuria on repeat urinalyses are upstaged to intermediate risk and thus warrant cystoscopy and renal ultrasound. High-risk patients (e.g., advanced age, >60 years; gross hematuria, >25 RBCs/HPF; >30 pack-years of smoking) warrant cystoscopy and computed tomography with urography (CTU) [19]. Despite these updated recommendations for hematuria workups, the utilization of updated guidelines may lag in clinical practice. Further studies are warranted to investigate the practice of updated hematuria guidelines at VA facilities.

**Figure 1:**
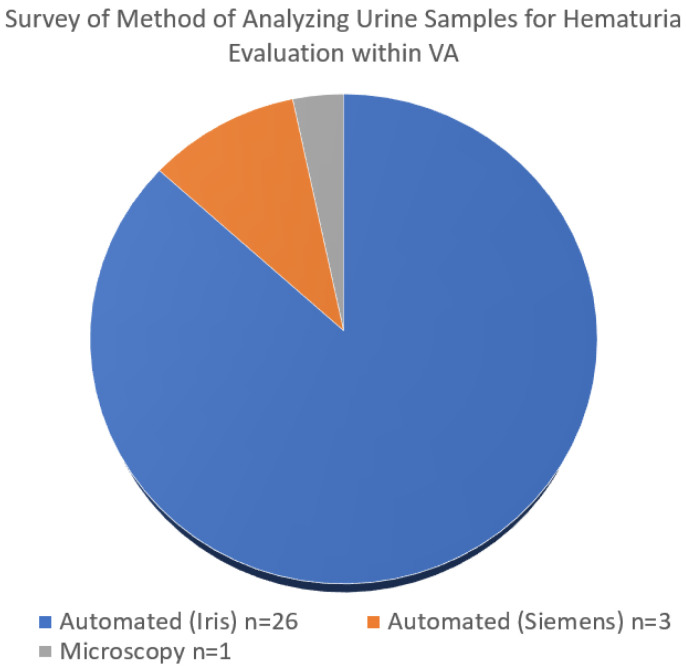
Surveyed of method of analyzing urine. Data for the figure was collected from urinalysis records in the VA electronic medical record.

The preparation of samples is another known challenge in the workup of AMH. While the AUA guidelines recommend a manual count, the automation of urine sediment examination may decrease inter-individual variation and reduce laboratory workloads [20]. A survey of VA pathologists found that of the 30 VA facilities polled across the country, 29 used automated cell counters to perform microscopic analyses (Figure 1). There is an opportunity for standardization within the VHAS that is veteran-centric and cost-effective.

Despite the guidelines, many patients with AMH do not get referred to urologists for evaluation [21,22]. Studies into referral patterns found that 48% of patients with detectable AMH had no further evaluation [21]. Despite all patients having justification for evaluation, only 12.8% (16% in VA institutions) were referred for cystoscopic evaluation, only 9% for cytology, and 30% for imaging [21]. There are no studies that explore the rationale for this pattern of behavior, but it is possibly related to the perceived low yield of cancer in patients with AMH—over 60% of cases evaluated have no explanation for the AMH so clinicians may feel there is little benefit to referring patients for expensive consultation and imaging [23].

### 2.2. Choice of Imaging Technique

Although a CTU is the recommended “gold standard” imaging modality, many veteran patients have renal insufficiency, which precludes the use of intravenous contrast. The risk of contrast-induced kidney injury can be as high as 25% in elderly patients with a combination of chronic kidney disease, diabetes, heart failure, and exposure to nephrotoxic drugs [24]. Renal protection protocols must be implemented for patients with reduced creatinine clearance.

Furthermore, the cost of CT is very high, while its yield for detecting malignancy is relatively low [25]. With the integration of ultrasonography into the hematuria algorithm, healthcare expenses have significantly been reduced without impacting the diagnostic yield or reducing the detection of malignancy [26]. For patients with renal impairment, in particular, renal and bladder ultrasounds are less invasive and less costly, avoid radiation exposure, and can be used to identify gross lesions without worsening the underlying renal disease [27]. In centers where imaging modalities are limited, routine ultrasound may be a better use of resources, as has been recommended for initial screening in many European countries [28].

One advantage of the VHAS is its comprehensive EMR. For other large referral centers, patients may present to new providers with limited access to prior records, including radiographic films and cystoscopic images. However, in the VHAS, the EMR allows for the easier identification of previously performed imaging and endoscopic studies, which can help delay the initiation of treatment.

## 3. Patient Management

### 3.1. TURBT and Re-Resection

For patients with high-risk NMIBC, the guidelines advise a repeat resection within six weeks to assess the completeness of the resection and to obtain a deeper specimen, including the muscularis propria. However, the wording of the AUA guidelines leaves room for different interpretations:

1. For high-grade T1 tumors, the AUA recommendation is that: “…the clinician should performing repeat transurethral resection of primary tumor…” [Level B recommendation] [13].
2. For high-risk, high-grade Ta tumors, the AUA recommendation is that: “…the clinician should consider performing repeat transurethral resection of primary tumor…” [Level C recommendation] [13].

While these cases may be sufficiently high-risk to warrant a repeat resection, a second TURBT is often not carried out due to resourcing or patient frailty. Furthermore, due to limited operating room availability, the timing of the repeat bladder tumor resection may not be within the 6-week window of the initial resection. Although a repeat resection is recommended, many centers worldwide fall short, with reported rates from 26% to 67% in various countries [29]. In the case of patients with high-grade Ta disease, residual tumor can be found at a repeat resection in up to 50% of patients, with 15% of these tumors being up-staged [13]. Large and multifocal tumors are at particular risk for incomplete resection at the initial TURBT. Despite the level C recommendation for the re-resection of high-risk, high-grade Ta tumors, approximately 25% of these patients underwent a repeat resection, demonstrating higher rates of muscle invasion and the need for radical cystectomy than those without repeat resections [30]. Factors contributing to the non-adherence to AUA guidelines, other than the ambiguous interpretation of the recommendations, include patient refusal, anesthetic risks, healthcare costs, and limited endoscopic resources.

As noted, repeat TURBT procedures can also place a strain on VA operating room resources. Typically, each VA medical center has a designated block time for urology in the operating room, with limited capacity to accommodate additional patients in the NMIBC guideline time frame. However, the argument for doing so is overwhelming, as incomplete resection is widely acknowledged to be a significant factor in so-called early recurrence [13]. Complete resection of the tumor, often with a staged intervention, and the appropriate acquisition of the muscularis propria are essential for adequate staging and effective clinical management.

### 3.2. Use of Intravesical Therapies

The intravesical instillation of chemotherapy within 24 h of resection has been shown to reduce the recurrence of NMIBC [13]. Traditionally, mitomycin C (MMC) has been the agent of choice, but newer randomized cooperative group data support the use of intravesical gemcitabine, which has a lower toxicity risk [31].

For intermediate-risk patients who do not have access to or who cannot tolerate intravesical chemotherapy, continuous flushing with saline irrigation for a period of up to 24 h may be as effective as MMC, with fewer side effects and a lower cost [32]. Compared to NMIBC patients without post-operative continuous bladder irrigation, those with overnight continuous bladder irrigation had lower recurrence rates [33].

Adjuvant intravesical immunotherapy with bacillus Camette–Guerin (BCG) has been a standard treatment for high-grade and carcinoma in situ (CIS) NMIBC (intermediate- and high-risk patients) for over 30 years, with confirmed efficacy [32,34]. Despite this, 40–45% of patients are found to have residual or recurrent tumor after initial BCG therapy, 20% of which are unresponsive [35].

### 3.3. Maintenance Intravesical Therapy Following Complete Response

For intermediate-risk patients who completely respond to an induction course of either chemotherapy or immunotherapy, there is a conditional recommendation to utilize maintenance therapy. In intermediate-risk patients, a one-year course of maintenance therapy BCG is supported by SWOG 8507 and European Association of Urology guidelines, but approximately 70% of patients who receive BCG will complain of side effects and 8% of these will discontinue treatment as a result [13,27,36].

The AUA recommends the induction and maintenance instillation of chemotherapy or immunotherapy for intermediate-risk NMIBC and BCG for high-risk NMIBC [13]. Especially in times of BCG shortage, gemcitabine has a limited evidence base but is potentially a useful addition to the treatment of recurrent urothelial carcinoma [37]. Many small observational studies have reported a reasonable response (40–60%) in NMIBC, including for recurrence tumors [32]. Furthermore, gemcitabine was found to be superior to MMC in efficacy and less toxic [37,38]. In patients with intermediate-risk NMIBC, gemcitabine had lower adverse effects compared to BCG and lower recurrence in BCG failure cases [39]. A Phase II study suggested there was a significant improvement in disease-free survival and lower recurrence rate with gemcitabine in patients who had failed initial BCG therapy, and a retrospective analysis reported that gemcitabine was well tolerated, with fewer side effects than BCG: 7% vs. 44% [39]. This may be advantageous for the VA population, where the tolerance of side effects is low and access to BCG is limited, especially during shortages. In times of BCG shortage, the AUA has recommended several strategies: (1) the utilization of intravesical chemotherapy for intermediate-risk NMIBC, (2) the prioritization of BCG for high-risk NMIBC patients, (3) the consideration of reduced dosage to 1/2 to 1/3 of BCG instillations, and (4) the utilization of alternative intravesical chemotherapy such as gemcitabine with docetaxel, valrubicin, and gemcitabine with MMC [40].

There is a substantial cost difference between the use of intravesical MMC and gemcitabine. As per Medicare, the average cost per dose for intravesical MMC and gemcitabine is USD 1152 and USD 65, respectively [41,42]. With the frequent instillation of chemotherapy, the financial toxicity of MMC for NMIBC is substantial compared to the alternatives.

**Figure 2:**
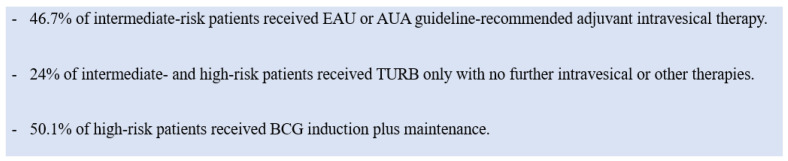
Practice patterns of intravesical treatment of intermediate- and high-risk patients. The data for this figure are from Mossanen et al. 2018 [44].

Despite the evidence, an international practice pattern survey found that intravesical therapy was underutilized (Figure 2) [43]. There are little published data on the reasons for its underutilization, but some speculate that hospital policies may impact its usage rates [44]. The administration of MMC requires coordination between pharmacists, the approval of Occupational Safety and Health officers, and proper equipment, meaning that institutions with limited resources may be unable to provide MMC, despite physician preferences. Nearly half of the hospitals in one study did not administer MMC [44].

### 3.4. Radical Cystectomy in NMIBC

Radical cystectomy is a highly complex surgical procedure that can significantly impact a patient’s quality of life. Consequently, radical cystectomy in the context of NMIBC is generally reserved for patients with high-risk or treatment-refractory disease. According to the 2020 AUA NMIBC guidelines, a clinician should not perform a radical cystectomy on low- or intermediate-risk disease unless other bladder-preserving treatments have failed. It should only be considered for patients whose disease presents variant histology, for a surgical candidate with high-grade T1 disease following a single course of induction intravesical BCG, or for high-risk patients with recurrent disease following two cycles of BCG or BCG maintenance therapy [45].

## 4. Using New Technologies to Improve Patient Management

**Figure 3:**
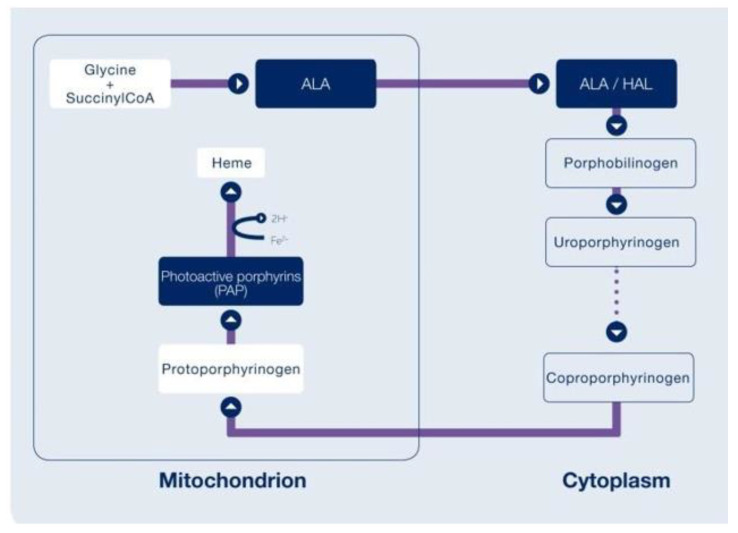
Hexaminolevulinate (HAL)’s mechanism of action. This diagram was sourced from the Cysview Photocure Product Information Fact Sheet.

**Figure 4:**
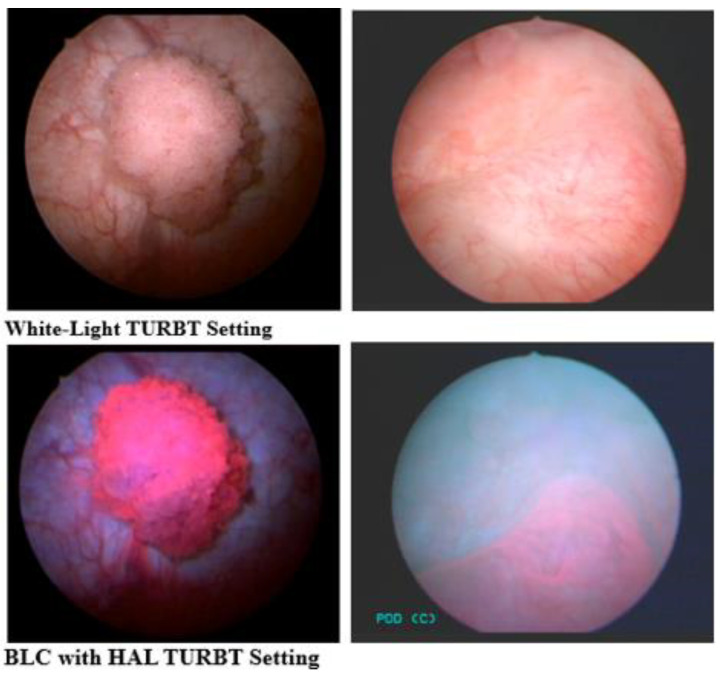
Comparison images of WLC versus BLC with HAL, using intraoperative photos taken by the authors.

Blue-light cystoscopy (BLC) using hexaminolevulinate (HAL) to enhance the visualization of tumors has recently become available (Europe 2005; USA 2010) and can be used in the operating room and for surveillance cystoscopy. HAL is an optical imaging agent that is instilled into the bladder and is then metabolized by enzymes in the heme synthetic pathway, resulting in the preferential accumulation of photoactive porphyrins in the more metabolically active cancer cells (Figure 3). Protoporphyrin IX fluoresces when exposed to blue light at wavelengths between 360 and 450 nm and emits red light in response. In the bladder, healthy tissue appears blue and malignant tissue will fluoresce red (Figure 4).

There is a strong evidence base for BLC, including six Phase III studies containing a total of more than 2100 patients, demonstrating the significantly increased detection of Ta and T1 tumors compared to white-light cystoscopy (WLC) alone [46,47,48,49,50,51].

### 4.1. BLC for Detection

The basis for BLC’s FDA approval was a study by Stenzl et al., which demonstrated that 16% of patients had additional Ta or T1 bladder tumors detected with BLC [46]. Additional tumors were high-grade in 43% of these patients. There was no significant difference between BLC and WLC in terms of false positive results [46]. Hermann et al. demonstrated that BLC with HAL could find residual lesions following an initial WL TURBT in up to 49% of patients [47].

A meta-analysis of data on over 2200 patients from nine studies confirmed there was a significant improvement in Ta and T1 tumor detection in 24.9% of patients and in CIS detection in 26.7% of patients, noted only through BLC with HAL [52].

### 4.2. BLC for Recurrence and Progression

The impact on recurrence was also significant, with a reduction in the recurrence rate of 10.9% at 12 months for patients treated with BLC [52]. A further study in which patients had a follow-up cystoscopy at 3, 6, or 9 months found that the time to recurrence was prolonged by 7 months in patients previously treated with BLC [53]. The same study reported a trend towards a decrease in the cystectomy rate [53]. A meta-analysis of five studies and 1301 patients found a significant reduction in the rate of progression over a period of at least two years following the use of BLC: 6.8% compared to 10.7% in WLC patients [46]. A re-analysis of data published in an earlier study (Stenzl 2010) found that the time to progression was prolonged [54]. The re-analysis was carried out according to a new IBCG definition, where additional indicators of progression were included.

A national registry has been set up to collect data on the use and benefit of BLC. So far, 275 VA patients have been enrolled nationwide, with 82 patients entered from the Michael E DeBakey VA Medical Center. An analysis of this cohort found that BLC improved detection, especially of flat lesions [55]. False positives were found to be influenced by any process that causes acute or chronic tissue inflammation, including recent infection or BCG administration. Inflammatory changes may be higher in the VA population than the general population due to the bladder cancer population being largely male, with BPH, incomplete bladder emptying, a higher rate of indwelling catheters, and asymptomatic bacteriuria.

BLC may offer advantages in the management of VA patients with NMIBC. As many patients are not eligible to, or do not, receive immediate intravesical chemotherapy at the time of their TURBT, the removal of all residual tumor is imperative for local control. Furthermore, the initial tumor resection must be thorough, since there are capacity constraints for repeat resections. Improved detection rates could also improve resource utilization. If an additional two out of ten patients were diagnosed earlier, the impact on recurrence and progression for those patients and the cost saving to the system are potentially significant.

## 5. A Case Study for the Implementation of New Technologies

The Michael E. DeBakey VA Medical Center was among the first to adopt BLC and has developed several standard operating procedures and certifications to streamline the process in the OR. Weekly meetings of the urologic oncology group determine whether upcoming TURBT procedures should include BLC, the type of anesthesia, and the requirement for upper tract imaging. If BLC is to be used, the HAL is instilled via a catheter one hour before the procedure by a preoperative-area nurse. Its use is also being considered for each veteran undergoing surveillance cystoscopy in the clinic.

For VA centers acquiring BLC for surveillance, the standard of care is cystoscopy in the clinic environment. This procedure can be performed safely given standardized clinic conditions, which include cleanliness and air exchanges. An adequately sterile technique, sterile processing of the cystoscopic equipment, and a sterile irrigation solution are required, as per VA regulations and standard operating procedures, to minimize the risk of infection.

Flexible cystoscopy of the lower urinary tract, similar to bronchoscopy and upper GI endoscopy, is not an intra-cavitary procedure. Therefore, a sterile environment, like the operating room, is not necessary for safe practice [56]. The necessary equipment for efficient cystoscopy includes at least two WLC+BLC-enabled towers and four flexible scopes, making it possible to perform up to eight surveillance cystoscopies per day, with instillations and cystoscopies being staggered. It is recommended that HAL be instilled with in-and-out catheterization.

Streaming video technology at the Michael E. DeBakey VAMC has allowed a very busy procedure clinic to be staffed by 2–3 physician assistants, a resident, and an attending in a supervisory role. The attending is able to watch four procedure rooms simultaneously; two-way communication is carried out using overhead webcams and images can be seen from any computer terminal [57]. Still images are saved to store in the patient’s chart in Vista Imaging and additional videos can be captured for reference. On average, 15–20 cystoscopy procedures can be carried out in a daily session, along with 10–15 prostate biopsies.

Through the VA EMR, we have developed a streamlined and detailed hematuria algorithm to provide information about the nature of the hematuria and appropriate imaging work-up. To prevent delays in care and losing patients to follow-up, patients with verified gross or microscopic hematuria are directly scheduled into the cystoscopy clinic rather than undergoing an intermediate consult visit.

Future plans include meeting with hardware suppliers to develop a standardized system across all tier-one VA medical centers, with the potential for cost savings and the standardization of care. Additionally, the Urological Society for American Veterans has a website where urologists and allied providers can participate in active discussions about the issues most relevant to the urologic care of veterans [58].

## 6. Summary

Veterans using the VHAS tend to be disadvantaged in terms of their sociodemographic and health status. Along with a higher comorbidity burden than non-veterans and resourcing allocations within the VHAS, the management of BC can be an arduous task. The integration of recent changes to the hematuria guidelines could potentially reduce the overutilization of resources at the VA without impacting the care of urologic patients. Although a repeat resection following an initial TURBT is strongly recommended for select high-risk patients, the frailty of the VA population and the available resources often limit the centers’ ability to carry out a second procedure. Given these constraints, BLC with HAL can improve the detection of bladder cancers and the thoroughness of resection relative to white-light cystoscopy. The VHAS presents unique challenges to bladder cancer patients but also opportunities to standardize practices across a healthcare system using innovative and cost-effective strategies.

## Figures and Tables

**Table 1:** ASA score comparison. ^1^ The cohort of patients scheduled for surgery was drawn from the 2012 ACS NSQIP database. Adapted from Table 1 in Hackett et al. [11].

	Cohort from General Population (% Total) ^1^	GU Patients Houston VA (% Total)	GU Patients National (% Total Only)
ASA PS 1	223,215 (9.7%)	5 (0.6%)	2.0%
ASA PS 2	1,055,582 (45.9%)	110 (12.6%)	22.2%
ASA PS 3	873,734 (38.0%)	593 (67.9%)	66.2%
ASA PS 4	139,302 (6.0%)	163 (18.7%)	6.3%
ASA PS 5	5796 (0.2%)	1 (0.1%)	0.0%
Missing	0 (0.0%)	1 (0.1%)	3.1%
Total	2,297,629	873	

## Data Availability

No new data were created or analyzed in the study. Data sharing is not applicable to this article.
